# Flexural Fracture Behavior and Mechanical Properties of SAP-PVA Fiber-Reinforced Concrete

**DOI:** 10.3390/ma19010203

**Published:** 2026-01-05

**Authors:** Xiaozhu Hu, Yanjun Wang, Faxiang Xie, Wenhao Cao

**Affiliations:** 1 CCCC Third Harbor Engineering Co., Ltd., China Communications Construction Company Ltd., Nanjing 210011, China; 2 College of Civil and Transportation Engineering, Hohai University, Nanjing 210098, China; 231304040001@hhu.edu.cn

**Keywords:** SAP, PVA fiber, internally curing concrete, fracture energy, fracture toughness, residual tensile strength, SEM microscopic analysis

## Abstract

To investigate the fracture behavior of super-absorbent polymer (SAP) internally cured polyvinyl alcohol (PVA) fiber-reinforced concrete (SAP-PVAC), three-point bending tests were carried out. This study systematically examined the effects of (1) PVA fiber content and (2) initial crack-depth-to-beam-height ratios (*a*_0_/*D*) on the failure modes, fracture toughness (*K*_IC_), and residual flexural tensile strength (*f*_R,1_) of SAP-PVAC beams. The test results demonstrate that SAP particles have a weakening effect on concrete strength (reduce about 6%). Still, the addition of PVA fibers can effectively improve the crack-resistance performance of SAP-PVAC and significantly increase the residual flexural tensile strength by 4.5–42%. The softening performance of the concrete is affected by the initial crack-height ratio. An increase in *a*_0_/*D* leads to an obvious increase in the crack opening displacement but has little impact on the fracture toughness, while the fracture energy shows a downward trend. SEM microscopic analysis reveals that the synergistic effect of SAP and PVA fibers exhibits a positive promoting effect on the toughening and crack resistance of SAP-PVAC specimens. These results establish a theoretical framework for SAP-PVAC fracture assessment and provide actionable guidelines for its shrinkage-crack mitigation structure engineering applications.

## 1. Introduction

As the most widely used material in infrastructure [1,2,3,4], concrete offers numerous advantages in engineering. However, its inherent brittleness and tendency to crack are key factors restricting its extensive application. The generation and propagation of cracks are common phenomena in concrete structures, which not only affect the mechanical properties of the structure but also significantly reduce its durability and service life [5]. Therefore, in-depth research on the fracture mechanism and post-crack softening behavior of concrete can not only provide a scientific basis for improving its crack resistance and durability but also has high practical application potential.

Super-absorbent polymer (SAP), as an efficient internal curing agent for concrete, can significantly reduce the early autogenous shrinkage of concrete. Its unique water absorption and expansion mechanism can slowly release the stored water during the cement hydration process, further promoting the cement hydration reaction and suppressing the formation of micro-cracks, effectively addressing the issue of poor internal post-curing effects in concrete [6,7]. Yao et al. [8] found that adding SAP to ECC (engineered cementitious composites) can improve the later-stage strength and toughness, with the tensile strain capacity increased by 92%. Ding et al. [9] believed that SAP can greatly improve the freeze–thaw resistance and impermeability of concrete, and SAP is more beneficial to the improvement of durability under dry-heat conditions. Xia et al. [10] demonstrated that SAP incorporation can effectively mitigate drying shrinkage in alkali-activated concrete (AAC), achieving shrinkage reduction rates ranging from 12.4% to 32.7% at 56 days. The optimal performance was observed at SAP and nano-silica contents of 0.2% and 2%, respectively, where AAC exhibited superior mechanical properties coupled with enhanced shrinkage suppression, which illustrates that the addition of SAP has a certain positive effect on concrete.

However, it should be noted that although SAP brings many benefits, excessive internal curing water intake and the pores left in the concrete after SAP loses water and shrinks may have an adverse impact on the strength of concrete [11,12]. Xie et al. [12] found in the research that the introduction of internal curing water and the pores left after SAP loses water and shrinks weaken the tensile strength of concrete. Hasholt et al. [11] pointed out that when the amount of water added for internal curing is too large, the water absorption rate of SAP will be too high, resulting in a loss of concrete strength. Xia’s study [10], likewise, addressed this phenomenon. To effectively make up for this potential negative impact, introducing fiber composites has become a widely recognized strategy. Polyvinyl alcohol (PVA) fibers contain hydroxyl functional groups [13], which show good synergistic effects with SAP. Stable hydrogen bonds can be formed between the interface molecules of the two, significantly enhancing the surface bonding strength between the fibers, matrix, and aggregates, thus improving the overall performance of the concrete [13,14,15]. Liu’s study [16] also revealed that for steel–PVA hybrid fiber-reinforced concrete (HFRC), increasing PVA fiber content reduces the proportion of steel fibers aligned at 90° while significantly enhancing both porosity and pore connectivity in HFRC. Although PVA fiber incorporation improves HFRC’s ductility, excessive fiber content negatively impacts strength parameters. In a separate investigation on 3D-printed PVA fiber-reinforced concrete, Liu et al. [17] demonstrated that fracture energy exhibits continuous growth with increasing fiber content, accompanied by progressive improvements in both crack initiation toughness and fracture instability toughness. Li [14] found through three-point bending tests and AE characteristic analysis that PVA fibers significantly improve the crack resistance, bending performance, and toughness of concrete. The test results of Jiang Jin et al. [18] show that PVA fibers have good crack resistance and toughening effects, significantly improving the splitting tensile and deformation capabilities of concrete. These findings indicate the favorable toughening and crack-resistance effects of PVA fiber-reinforced materials. In addition, PVA fibers are an ideal environmentally friendly cement-reinforcing material, with sound physical and chemical properties such as alkali resistance and weather resistance. These excellent characteristics have led to the wide promotion and application of PVA fibers in practical engineering, making them one of the research hotspots in the field of current concrete material science.

At present, relevant scholars have conducted many studies on the effects of SAP or PVA fibers on the fracture performance of concrete. However, little attention has been paid to the influence of the synergistic effect of SAP and PVA fibers on the concrete flexural mechanical properties. Based on the background, this paper carried out three-point bending (TPB) tests on internal curing SAP PVA fiber-reinforced concrete (SAP-PVAC) specimens, analyzed the variation laws of the load–displacement curves (P-CMOD) of notched beams with different fiber contents and the post-crack softening characteristics, deeply studied the fracture energy and internal flexural fracture mechanism of fiber-reinforced concrete under TPB loads, and explored the toughening and crack resistance mechanism of the synergistic effect of SAP-PVAC from a microscopic perspective through scanning electron microscope (SEM) analysis. The research results can provide a theoretical reference for the engineering application and damage detection of SAP-PVAC.

## 2. Experimental Program

### 2.1. Experimental Materials

**Figure 1:**
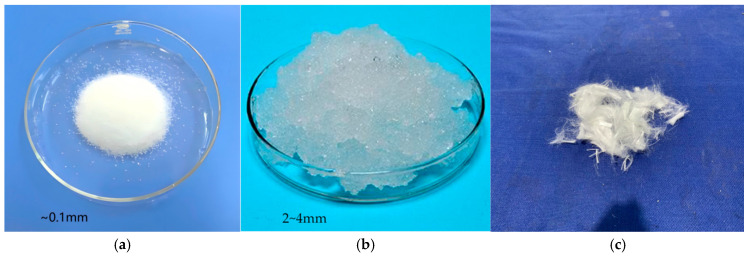
SAP particles and PVA fiber used in the experiments. (**a**) Dry SAP particles. (**b**) Saturated SAP particles. (**c**) PVA fiber.

The cement used in the experiment is P.O. 42.5 Normal Portland cement. The fine aggregate is natural river sand with an average particle size of 0.35–0.5 mm and a fineness modulus between 2.3 and 3.0. The mixing water is ordinary tap water, and the coarse aggregate is 5–20 mm continuously graded basalt crushed stone. A composite polycarboxylate high-performance water-reducing agent is added to the concrete to improve its fluidity. High-strength and high-elastic-modulus PVA fibers and synthetic-based SAP sodium acrylate with an average particle size of 100 μm are selected. This SAP material has excellent water absorption, water release properties, and the characteristic of enhancing the bonding strength of the interfacial transition zone [19,20,21]. Figure 1 shows the pictures of SAP and PVA fibers, and their dimensions and physical parameters are shown in Table 1 and Table 2.

### 2.2. Sample Preparation

The experiment adopts the mixing proportion of C30 concrete (according to the Fib Model Code 2010 [23]). On this basis, the mixed proportions of SAP-PVAC and its control group are designed, and different volume contents of PVA fibers are set, which are 0%, 0.05%, 0.1%, 0.15%, and 0.2%, respectively. Studies by Zhang [24] and He et al. [25] revealed that additional water introduction would affect the mechanical properties of SAP-modified concrete. Currently, the theoretical calculation method for determining the optimal internal conservation water quality in SAP principally follows Powers’ model [26]. Powers proposed that when the water-to-cement ratio (*W*/*C*) of concrete is below 0.42, insufficient hydration occurs internally, thus necessitating internal curing water introduction to ensure complete cement hydration. The Powers model [26] is shown by Equation (1):

(1) $$(W_{ic}/C)=\left\{\begin{array}{c} 0.18(W/C),(W/C)\leq 0.36\\ 0.42-(W/C),0.36\leq (W/C)\leq 0.42 \end{array}\right.$$

where *W*/*C* is the original water-cement ratio of the concrete, and *W*_ic_/*C* is the internal curing water-cement ratio. In this paper, the designed concrete *W*/*C* is 0.323. According to Equation (1), the optimal *W*_ic_/*C* is determined to be 0.058. Therefore, the internal conservation water demand can be determined as 27.9 kg/m^3^ based on the unit cement content. The research of relevant scholars [27,28] revealed that the water absorption ratio of SAP particles is approximately 24.90 g/g in tap water, while its optimal absorption ratio in cement paste solution reaches 25 g/g. Consequently, this study adopted a pre-absorption ratio of 25 g/g for SAP particles in cement paste solution. Based on these parameters, the content of SAP particles in SAP-PVAC specimens was calculated as 1.116 kg/m^3^. The specific mix proportions of the concrete are shown in Table 3.

The initial crack-height ratio (*a*_0_/*D*) is a critical parameter governing the fracture behavior of concrete beams. Wu et al. [29,30] observed that the initiation fracture toughness stabilizes when *a*_0_/*D* exceeds 0.4, while the unstable fracture toughness shows negligible correlation with *a*_0_/*D*. To investigate fracture evolution in the subcritical crack-growth regime, this study tests three normalized crack-height ratios (*a*_0_/*D* = 0.2–0.35), supplementing existing data for *a*_0_/*D* < 0.4, where nonlinear fracture mechanics dominate.

In this experiment, five groups of concrete specimens with different mix proportions are designed, including the control group C and four groups of SP-0.05% to SP-0.20% with different PVA fiber contents. For each mix proportion, three different initial crack heights (2 cm, 3 cm, and 5 cm) are designed, and three notched beam parallel specimens are cast, respectively (a total of 45 specimens). The size of each specimen is 150 × 150 × 550 mm. The ratio of the span to the beam height is 3.33, and the initial crack depth to beam height (*a*_0_/*D*) is 0.133, 0.200, and 0.333, respectively. To ensure the test accuracy, a high-precision cutting machine is used to cut an initial notch with a thickness of 5 mm before the test.

**Figure 2:**
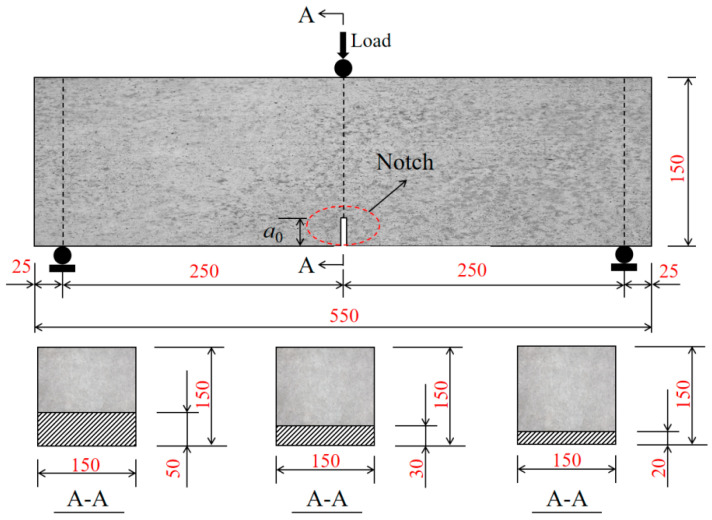
Specimen dimensions and loading arrangement (unit: mm).

The specimen numbering rule is as follows: C-*a*_0_/*D* represents an ordinary concrete beam; SP-*p*%-*a*_0_/*D* represents an internal curing fiber-reinforced concrete beam, where *p*% represents the PVA fiber content, and *a*_0_/*D* represents the initial crack-height ratio. The specific dimensions and loading arrangements of the specimens are shown in Figure 2.

### 2.3. Experimental Methods

According to the specification fib Model Code 2010 [23], the compressive strength of concrete is tested. Standard cube specimens (150 × 150 × 150 mm^3^) are used for a 28-day compressive strength test, and the average compressive strength *f*_cm_ of ordinary concrete is measured to be 43.06 MPa, with a standard deviation of 1.04 MPa.

**Figure 3:**
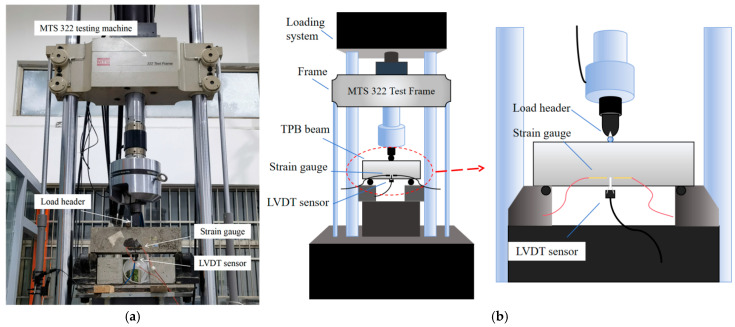
Loading and fracture schematic of the test setup. (**a**) Test device. (**b**) Load diagram.

The loading system adopted in this study was an MTS 322.41 T-flange test frame equipped with electro-hydraulic servo actuators. The MTS 322.41 testing machine was manufactured by MTS Systems Corporation (a U.S.-based provider headquartered in Minneapolis, MN, USA). With a load capacity of 500 kN (the load measurement accuracy exceeds 0.5%) and fatigue testing capability (0–30 Hz), the apparatus conducted mode I three-point bending fracture tests on SAP-PVAC beam specimens under ambient temperature conditions. The test process strictly follows the specifications RILEM TC 162-TDF [31,32] to observe the complete fracture process of the specimens at the initial crack. At the same time, to measure the cracking time and cracking load of the specimens, strain gauges are pasted at the tip of the initial crack of the specimens. During the loading process, an extensometer installed under the specimen is used to control the loading process, where the loading rate is set to 0.001 mm/s. In addition, to measure the crack mouth opening displacement (*CMOD*), an LVDT (linear variable differential transformer) is installed near the prefabricated crack mouth, and a steel plate with a thickness of 3 mm is used to fix the clip gauge to ensure the accuracy of the measurement. Figure 3 is a schematic loading device and a diagram of the three-point bending (TPB) test loading. Due to instrumentation limitations, scanning electron microscopy (SEM) analysis was employed solely for visual characterization without quantitative measurements in the SEM analysis part. The SEM analysis was performed using a TESCAN MIRA4 field-emission scanning electron microscope (TESCAN, Brno, Czech Republic).

## 3. Results and Discussion

### 3.1. Failure Modes

**Figure 4:**
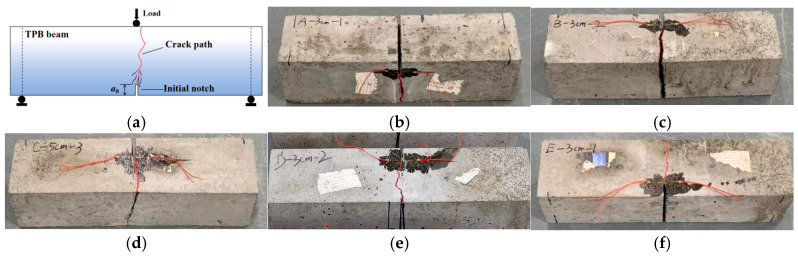
Crack failure modes of specimens with different fiber contents. (**a**) Theoretical failure mode of concrete. (**b**) Crack pattern of ordinary concrete C. (**c**) Crack pattern of SP-0.05% specimen. (**d**) Crack pattern of SP-0.10% specimen. (**e**) Crack pattern of SP-0.15% specimen. (**f**) Crack pattern of SP-0.20% specimen.

Figure 4 shows the failure modes of concrete specimens with different PVA fiber contents and the control specimen, intuitively reflecting the important influence of PVA fiber content on the fracture performance of concrete. As can be seen from the figure, the cracks all start from the top of the prefabricated notch and gradually penetrate the entire cross-section under the action of external loads. When the PVA fiber content is low (such as 0% or 0.05%), the crack width is large, indicating that the reinforcement effect of the fibers is limited. However, as the fiber content gradually increases, the crack opening width decreases, and the crack distribution becomes more compact. At the same time, the crack propagation angle also increases. This is because when the fiber content is low, the interfacial bonding force and aggregate interlocking ability are weak, resulting in cracks being more likely to concentrate and develop rapidly and vertically during the propagation process. As the PVA fiber content increases, the fibers form a more effective network in the concrete, which can more effectively disperse the load stress, thus suppressing the concentrated propagation of micro-cracks. In addition, the fibers also provide interfacial bonding and bridging effects, further slowing down the crack propagation speed and scale. These positive effects jointly cause the cracks to develop inclined along the minimum energy consumption path, so the propagation angle would also deviate from a vertical line.

**Figure 5:**
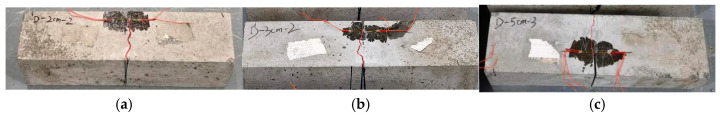
Crack patterns of SP-0.15% specimens at different initial crack-height ratios. (**a**) Crack pattern of SP-0.15%-0.133 specimen. (**b**) Crack pattern of SP-0.15%-0.200 specimen. (**c**) Crack pattern of SP-0.15%-0.333 specimen.

Figure 5 illustrates the failure modes of concrete specimens with different initial crack-height ratios. As can be seen from the figure, although the crack width does not change significantly among different specimens, the specimens with a larger initial crack length have a smaller crack propagation angle, which reflects the influence of the initial crack length on the local constraint conditions at the crack tip. When the initial crack length is larger, the local constraint at the crack tip will be correspondingly reduced, resulting in a more concentrated and single energy-release path during the crack propagation process. Therefore, the crack will be more inclined to propagate along the shortest path, resulting in a straight crack. On the contrary, for specimens with a smaller initial crack length, the crack tip will be more constrained, and the energy-release path will be more diverse. This prompts the crack to choose a more complex and twisting path during the crack propagation.

### 3.2. P-CMOD Curves

For notched specimens, under the three-point bending load, according to the crack propagation process, the load–crack mouth opening displacement (P-CMOD) curve can be divided into three stages: I. crack-initiation stage; II. stable crack propagation stage; III. unstable crack propagation stage. These three stages represent different development states of the crack, from initial formation to final failure. Point A and B demarcate the three-phase transition of flexural fracture behavior, the point A is initial cracking point, and the point B is critical cracking point.

**Figure 6:**
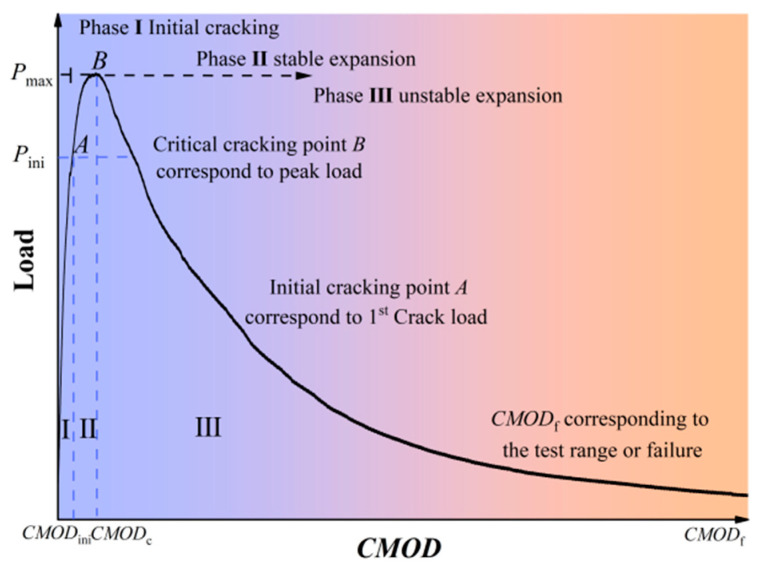
The prominent characteristics of the P-CMOD curve.

Figure 6 shows the prominent characteristics of the P-CMOD curve of SAP-PVAC beam specimens. In the crack-initiation stage, as the load gradually increases, micro-cracks begin to form inside the specimen and gradually converge into the main crack, and the P-CMOD curve rises simultaneously. As the crack further propagates, the bearing capacity of the specimen reaches its peak value. Then, the crack expands rapidly until the specimen fails. In the crack propagation stage, the P-CMOD curve rises sharply until it reaches the peak load. As the specimen is damaged, its bearing capacity decreases rapidly, as well as the load decreases, and the CMOD increases. Then, the P-CMOD curve enters the descending section until the specimen is completely damaged.

**Figure 7:**
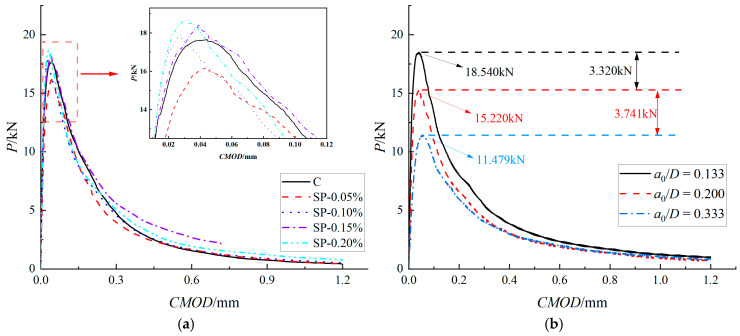
Typical P-CMOD curves of three-point bending fracture tests. (**a**) P-CMOD curves for different PVA contents, with $a_{0}/D=$ 0.133. (**b**) P-CMOD curves of SP-0.20% specimens at different initial crack-height ratios.

Figure 7 illustrates the typical P-CMOD test curves of specimens with different PVA fiber contents and initial notch-to-height ratios. As shown in Figure 7, the PVA fiber content has an enhancing effect on the peak load. As the fiber content gradually increases, the descending section of the curve becomes gentler, indicating that the addition of PVA fibers not only improves the bearing capacity of the specimen but also effectively delays the failure process of the specimen after reaching the peak load, enhancing the ductility of the specimen. The initial crack-height ratio has a significant impact on fracturing performance. As the initial crack-height ratio increases, the overall bearing capacity of the specimen significantly decreases, and the failure deformation decreases accordingly. The crack mouth opening displacement at the critical state is increased, which further reflects the influence of the size effect on the fracture performance.

According to the experimental curves, loads (*P*_ini_, *P*_max_), crack opening displacements (*CMOD*_ini_, *CMOD*_max_), flexural tensile strength *f*_t_, and elastic modulus *E* are obtained, as shown in Table 4.

**Figure 8:**
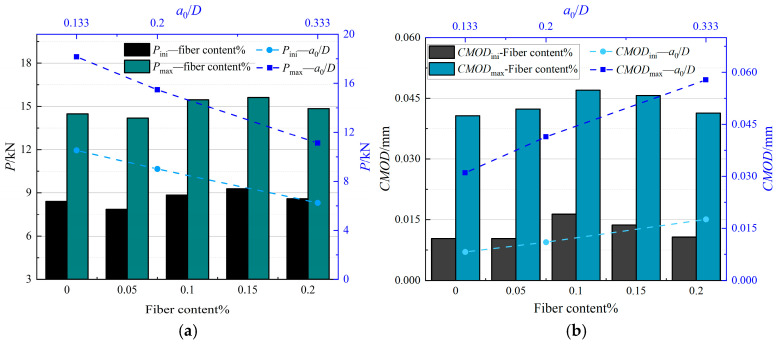
Load and *CMOD* characteristic values of SAP-PVAC with different fiber contents and initial crack-height ratios. (**a**) Initial cracking load and peak load. (**b**) Initial crack mouth opening displacement and critical crack mouth opening displacement.

Figure 8a reveals the variation laws of the parameters related to the initial and critical cracking points with the PVA fiber content. Compared with the control specimen, the incorporation of PVA fibers has a first-increasing-then-decreasing impact on the bearing capacity of the concrete specimens. Specifically, when the PVA fiber content is 0.05%, the average peak load (*P*_max_) almost remains constant from 14.478 kN to 14.184 kN, which could be treated as experimental error. As the fiber content increases to 0.15% content, the average peak load increases 7.89%. At the same time, as the PVA fiber content increases, the average crack mouth opening displacements (*CMOD*_ini_ and *CMOD*_c_) generally increase. The initial crack mouth opening displacement (*CMOD*_ini_) increases by 54.4%, from 0.011 mm to 0.017 mm. The critical crack mouth opening displacement (*CMOD*_c_) increases by 17.5%, from 0.04 mm to 0.047 mm. This variation can be attributed to the following aspects. At a low PVA fiber content, the improvement of the bearing capacity of the concrete by the fibers is limited. At the same time, the internal curing water introduced by the SAP particles may lead to an increase in the water-cement ratio and porosity of the concrete, thus reducing the peak load of the specimen. However, as the fiber content increases, the network structure formed by the fibers in the concrete begins to play a significant role, enhancing the interfacial bonding and bridging effects and improving the toughness and ductility of the concrete. In addition, the fibers can also make the crack distribution more uniform through the energy dissipation mechanism, thus delaying the crack propagation speed and increasing the crack mouth opening displacement.

Figure 8b shows the variation laws of the load and CMOD characteristic values of SAP-PVAC specimens. As can be seen from the figure, as *a*_0_/*D* gradually increases, Pini and Pmax of the specimen both show a significant downward trend, with decreases of 40.7% and 38.8%, respectively. This change indicates that an increase in the initial crack-height ratio hurts the bearing capacity of the specimen. At the same time, *CMOD*_ini_ and *CMOD*_c_ increase significantly with the increase in *a*_0_/*D*, with increases of 114.5% and 87.1%, respectively. This trend reflects the change in crack propagation behavior. When the initial crack-height ratio increases, the initial shape of the crack tip changes, and the effective bearing area decreases, resulting in a reduction in the bearing capacity of the specimen. The initial flexural stiffness of the concrete is also weakened, and the critical crack length increases, making it easier for the stress at the crack tip to concentrate and expand, thus manifested as a significant increase in the initial and critical crack mouth opening displacements. As shown in Table 4, the elastic modulus *E* (calculated by Equation (14)) demonstrates two distinct trends: (1) specimens with identical mix proportions show minimal variation (±10%) in *E* across different *a*_0_/*D* ratios, confirming geometric independence of pre-crack stiffness; (2) with increasing PVA fiber content (0–2.0 vol%), *E* follows a characteristic V-shaped curve. Two primary mechanisms underlie this phenomenon: (1) Inherent material variability (non-uniform component distribution, vibration-induced segregation) and experimental deviations (loading misalignment) may contribute to these observations. (2) More critically, the internal curing water from SAP water-absorbent polymers increases both water-to-binder ratio and porosity, thereby reducing composite stiffness. However, as PVA fiber content rises beyond 1.0%, their intrinsic high strength/modulus properties become dominant. The synergistic SAP-PVA system enhances crack bridging efficiency and restricts micro-crack propagation, ultimately restoring and surpassing the original stiffness (see microstructural evidence in Section 4).

### 3.3. Fracture Characteristics

#### 3.3.1. Fracture Energy and Fracture Toughness

Fracture energy (*G*_F_) is an important indicator for measuring the energy dissipation during crack propagation in concrete. It directly reflects the crack-resistance performance and energy-absorption capacity of the material and is one of the key parameters for evaluating the fracture performance of concrete [33,34]. The magnitude of the fracture energy is not only related to the constituent materials but also affected by various factors such as crack morphology and loading conditions. The formula for calculating the three-point bending fracture energy of concrete is [35]:

(2) $$G_{F}=\frac{W_{0}+mg\delta_{\max}}{A_{lig}}=\frac{\int_{0}^{\delta_{\max}}Pd\delta +mg\delta_{\max}}{t(D-a_{0})}$$

where *A*_lig_ is the fracture ligament area, *W*_0_ is the area under the load–displacement curve, (the fracture energy of specimens was calculated following *δ* = *L*/(4*H*)·*CMOD*, as recommended by RILEM TC 162-TDF [36]), *P* is the load, *mg* is the mass between the net-spans (including the loading mass), and *δ*_max_ is the maximum displacement in the experiment. *t* is the width of the beam, *D* is the height of the beam, and *a*_0_ is the initial notch length.

Fracture toughness (*K*_IC_) is an important parameter describing the stress state around the crack tip of a material under external loads [4,37]. It reflects the ability of the material to resist crack propagation and is an important manifestation of the material’s toughness. To more comprehensively describe the fracture performance of concrete, Xu and Reinhardt [38] proposed a double-K fracture model, which divides the fracture toughness into an initial cracking toughness parameter and an unstable fracture toughness parameter. The initial cracking toughness parameter characterizes the critical stress state when the concrete crack begins to expand, while the unstable fracture toughness parameter describes the upper limit of the development of stable cracks. Through these two parameters, the double-K fracture model can more accurately judge the crack propagation trend and stability of concrete, providing powerful theoretical support for the fracture performance evaluation and design of concrete structures. The double-K criteria are listed as follows in Equation (3):

(3) $$\left\{\begin{array}{l} K_{IC}<K_{\mathrm{IC}}^{\mathrm{ini}},\;\mathrm{cracks}\mathrm{don}’t\mathrm{break};\\ K_{\mathrm{IC}}^{\mathrm{ini}}\leq K_{IC}<K_{\mathrm{IC}}^{\mathrm{un}},\;\mathrm{stable}\;\mathrm{crack}\;\mathrm{propagation};\\ K_{IC}\geq K_{\mathrm{IC}}^{\mathrm{un}},\;\mathrm{unstable}\;\mathrm{crack}\;\mathrm{propagation}. \end{array}\right.$$

$K_{\mathrm{IC}}^{\mathrm{ini}}$ is the initial cracking toughness parameter, and $K_{\mathrm{IC}}^{\mathrm{un}}$ is the unstable cracking toughness parameter in Equation (3).

In the three-point bending fracture test of concrete, the span-to-height ratio of the standard specimen is usually set as 4 [30]. For non-standard specimens, the calculation formula for the standard specimen is not applicable [31,32]. Therefore, the linear interpolation method and equivalent elastic theory proposed by Yin et al. [31,33] can be used to calculate the relevant fracture parameters using the following equations:

(4) $$\sigma_{N}=\frac{3S}{2tD^{2}}(P+0.5mg)$$

(5) $$K_{\mathrm{IC}}=\sigma_{N}\sqrt{\pi a}F_{\beta }(a/D)$$

(6) $$CMOD=\frac{4\sigma_{N}a}{E_{c}}V_{\beta }(\alpha )$$

(7) $$\alpha =\frac{a+h}{D+h}$$

where *σ_N_* is the nominal stress, *P* is the external load, *S* is the span of beam, *t* is the width of beam, *D* is the height of beam, *F*_β_(*α*) is the geometrical factor for determining $K_{\mathrm{IC}}^{\mathrm{ini}}$, and *V*_β_(*α*) is the geometrical factor for determining *a*. *E_c_* is the Young’s modulus of concrete, *a* is the effective crack length, *α* is the relative crack length normalized based on beam height *D*, and h is the thickness of the blade for fixing the LVDT sensor.

For three-point bending specimens with a span-to-height ratio of 2.5 ≤ *S*/*D* ≤ 4, *V*_β_(α) and *F*_β_(*a*/*D*) can be determined by the linear interpolation method, as shown in Equations (8) and (9):

(8) $$V_{\beta }(\alpha )=\frac{\beta -2.5}{1.5}V_{4}(\alpha )-\frac{\beta -4}{1.5}V_{2.5}(\alpha )$$

(9) $$F_{\beta }(a/D)=\frac{\beta -2.5}{1.5}F_{4}(a/D)-\frac{\beta -4}{1.5}F_{2.5}(a/D)$$

where β = *S*/*D*, and *V*_2.5_(α), *F*_2.5_(*a*/*D*), *V*_4_(α) and *F*_4_(*a*/*D*) can be calculated by linear interpolation [39] following Equations (10)–(13):

(10) $$V_{2.5}(\alpha )=0.65-1.88\alpha +3.02\alpha^{2}-2.69\alpha^{3}+\frac{0.68}{{\left(1-\alpha \right)}^{2}}$$

(11) $$V_{4}(\alpha )=0.76-2.28\alpha +3.87\alpha^{2}-2.04\alpha^{3}+\frac{0.66}{{\left(1-\alpha \right)}^{2}}$$

(12) $$F_{2.5}(a/D)=\frac{1}{\sqrt{\pi }}\frac{1.83-1.65a/D+4.76{\left(a/D\right)}^{2}-5.3{\left(a/D\right)}^{3}+2.51{\left(a/D\right)}^{4}}{\left(1+2a/D\right){\left(1-a/D\right)}^{3/2}}$$

(13) $$F_{4}(a/D)=\frac{1}{\sqrt{\pi }}\frac{1.99-a/D(1-a/D)[2.15-3.93a/D+2.7{\left(a/D\right)}^{2}]}{(1+2a/D){\left(1-a/D\right)}^{3/2}}$$

The Young’s modulus *E_c_* and flexural tensile strength can be calculated by Equation (14):

(14) $$\begin{array}{l} E_{c}=\frac{6Sa_{0}}{BD^{2}C_{i}}V_{\beta }(\alpha_{0})\\ f_{t}=\frac{P_{\max}S}{t{\left(D-a_{0}\right)}^{2}} \end{array}$$

where *S* is the net-span of the beam, *a*_0_ is the initial crack length, α_0_ = (*a*_0_ + *h*)/(*D* + *h*), and the initial compliance *C_i_* is calculated from any point on the straight-line section of the rising part of the P-CMOD curve.

Substituting the peak load *P*_max_ and the critical crack mouth opening displacement *CMOD_c_* into Equation (6) can obtain the critical crack length *a*_c_, and it can also be calculated using the weight formula [34]. Therefore, for non-standard span-to-height ratio three-point bending specimens, when *P_ini_*, *P_max_*, and *a_c_* are known, $K_{\mathrm{IC}}^{\mathrm{ini}}$ and $K_{\mathrm{IC}}^{\mathrm{un}}$ can then be obtained by Equation (5).

**Figure 9:**
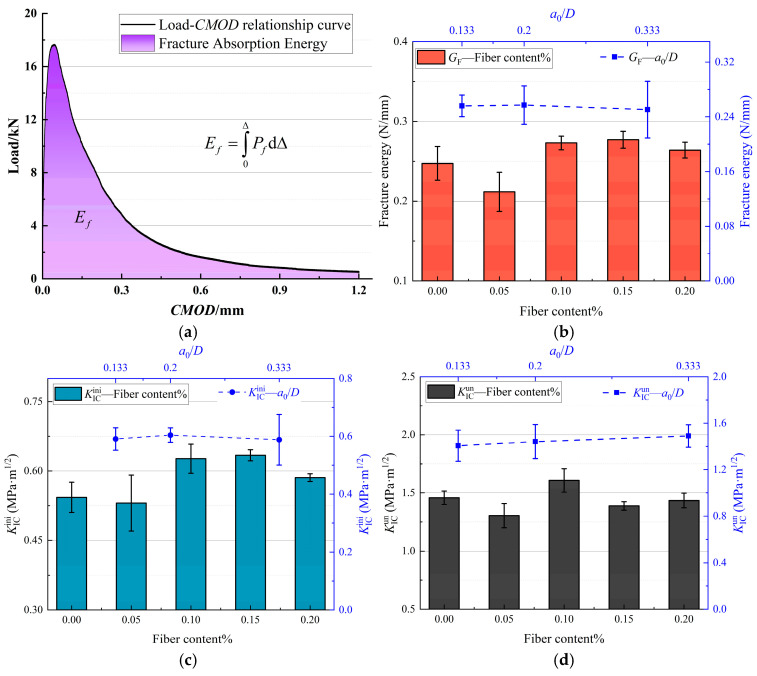
Fracture characteristics and parameters of SAP-PVAC under different fiber contents and initial crack-height ratios. (**a**) Fracture-absorbed energy *E*_f_. (**b**) Fracture energy *G*_F_. (**c**) Initial fracture toughness $K_{\mathrm{IC}}^{\mathrm{ini}}$. (**d**) Unstable fracture toughness $K_{\mathrm{IC}}^{\mathrm{un}}$.

According to the above formulas, the fracture energy and fracture toughness of the SAP-PVAC notched beam are determined, and the results are shown in Figure 9. The results show that the fracture energy and fracture toughness are not the result of a single-factor effect but a comprehensive manifestation of the combined action of SAP and PVA fibers. As the fiber content changes, both the fracture energy and fracture toughness show significant change trends, indicating that the fiber content has an important impact on the fracture performance of concrete. In contrast, the impact of *a*_0_/*D* on the fracture performance is not remarkable.

**Figure 10:**
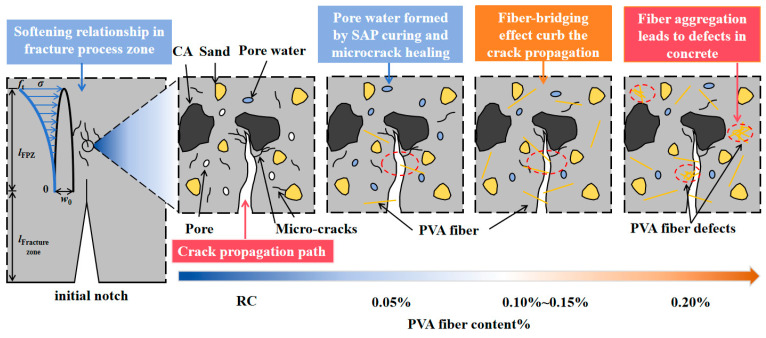
Mechanism of crack propagation in SAP-PVAC specimens with different fiber contents.

Figure 9 demonstrates that, in comparison to ordinary concrete, SAP-PVAC specimens exhibit enhanced fracture energy and fracture toughness with increasing PVA fiber content. However, a minor decrease is observed at 0.05% fiber content. The mechanism of this phenomenon can be further explained through Figure 10.

When SAP and PVA fibers are incorporated into concrete in the combination of SP-0.05%, the internal curing effect of SAP promotes the cement hydration reaction, which helps to reduce the formation of matrix micro-cracks. However, due to the increase in the water-cement ratio of concrete caused by the introduction of SAP, the number of capillary pores inside the concrete increases. At low fiber content, these capillary pores and micro-cracks easily become channels for crack propagation, and a small amount of PVA fibers is insufficient to effectively suppress the crack propagation. Therefore, the fracture performance of the specimen is lower than that of the control specimen. As the PVA fiber content increases to the range of 0.10–0.15%, PVA fibers, due to their high hydrophilicity, form a stable bond with the cement matrix, significantly enhancing the interfacial bonding performance.

In the fracture zone, PVA fibers exhibit an obvious bridging effect, effectively suppressing the crack propagation, dissipating the energy during crack propagation, thus improving the fracture performance of the specimen. When the PVA fiber content further increases to 0.2%, due to the high aspect ratio and strong hydrophilicity of the fibers, they easily form a hydration film in the concrete and adsorb and aggregate into clusters. This aggregation phenomenon not only reduces the contact area between the fibers and the cement matrix, reducing the interfacial bonding strength, but may also form defects and become new crack sources. Therefore, although the fiber content increases, the fracture performance of the specimen shows a certain degree of decline.

The fracture performance results of specimens with different mix proportions are shown in Table 5. Figure 10 demonstrates the influences of PVA fibers, SAP particles, and initial defects on the crack propagation mechanism of SAP-PVAC. By comparing concrete specimens with different initial crack-height ratios (*a*_0_/*D*), statistical analysis revealed that, except for SP-0.05% and SP-0.01%, the variation range (standard deviation) of fracture parameters (fracture toughness and fracture energy) for all other mix proportion specimens remained within 10% or even 5%. Furthermore, none of the tested mix proportions exhibited fracture parameter variations exceeding 15%. It can be concluded that $K_{\mathrm{IC}}^{\mathrm{ini}}$ and $K_{\mathrm{IC}}^{\mathrm{un}}$ do not change significantly with the variation in *a*_0_/*D*. This research result is consistent with the conclusions of existing studies [29,30], indicating that these two parameters are independent of the initial crack-height ratio. In addition, as the initial crack-height ratio increases, the effective load-bearing area of the specimen decreases, resulting in a reduction in the energy required for crack propagation. Therefore, the fracture energy of the specimen shows a decreasing trend with the increase in the initial crack-height ratio.

In summary, SAP-PVAC exhibits the best crack-resistance performance and fracture toughness in the range of PVA fiber content from 0.10% to 0.15%. Although the initial crack-height ratio has a certain impact on fracture performance, it is not a decisive factor.

#### 3.3.2. Residual Flexural Tensile Strength

When evaluating the limit state of SAP-PVAC [36] after the peak loads, the residual flexural tensile strength *f*_R,1_ is a crucial indicator, and it can reflect the influence of fibers on the descending section of the P-CMOD curve, thereby describing the morphological characteristics of the descending branch of the curve, which is calculated according to RILEM TC 162-TDF [32] and CEB-FIP Model Code 2010 [23]. It is also an important parameter for evaluating the energy dissipation, toughness, and post-cracking resistance performance of fiber-reinforced concrete. The higher the value, the more obvious the fiber-bridging effect, indicating that the fiber-bridging effect in the concrete is more obvious, which can better suppress the crack propagation and improve the overall performance of the concrete. Since the *CMOD*_max_ of SAP-PVAC concrete did not exceed 1.5 mm, this study selected *f*_R,1_ as the reference variable for residual flexural tensile strength. According to Model Code 2010 [23], the parameter *f*_R,1_ is evaluated from the P-CMOD relationship, as follows, Equation (15):

(15) $$f_{R,1}=\frac{3P_{1}S}{2Bh_{\mathrm{sp}}^{2}}$$

where *f*_R,1_ [MPa] is the residual flexural tensile strength corresponding to *CMOD* = *CMOD*_1_ (*CMOD*_1_ = 0.5 mm); *P*_1_ [kN] is the load corresponding to *CMOD* = *CMOD*_1_; *S* [mm] is the span length of beam; *B* [mm] is the specimen width; *h*_sp_ = *D* − *a*_0_ [mm] is the distance between the notch tip and the top of the specimen.

**Figure 11:**
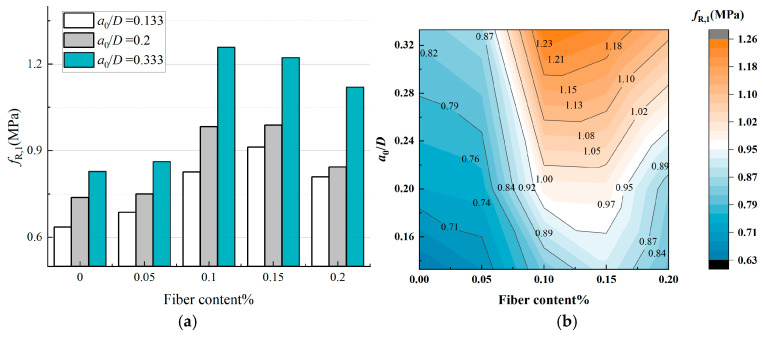
Residual flexural tensile strength of SAP-PVAC under different fiber contents and initial crack-height ratios. (**a**) Bar chart of residual flexural tensile strength. (**b**) Trend line of residual flexural tensile strength.

Table 5 and Figure 11 show *f*_R,1_ of SAP-PVAC specimens and its changing trend with the PVA fiber content and *a*_0_/*D*. As can be seen from Figure 11, *f*_R,1_ is affected by the parameters. When *a*_0_/*D* remains unchanged, as the PVA fiber content increases, *f*_R,1_ shows a significant upward trend. Notably, at a fiber content of 0.10%, *f*_R,1_ reaches its highest increase rate of 51.9%. This result indicates that the bridging effect of PVA fibers plays an important role in the stage of concrete failure and instability, enabling the concrete to maintain a certain strength when subjected to a large load. Furthermore, Figure 11 reveals that when the PVA fiber content exceeds 0.1% and reaches 0.2%, the *f*_R,1_ exhibits a declining trend, although it remains higher than normal concrete specimens. This observation aligns with the findings of Shen et al. [40], which reported that the flexural strength of concrete first increases and then decreases with rising PVA fiber content in 0.2%. This phenomenon may be attributed to the large aspect ratio of the PVA fibers used in the experiment, which are classified as long fibers with strong hydrophilicity. Their tendency to form hydration shells and aggregate into clusters could (1) reduce the contact area between fibers and the cement matrix and (2) decrease the number of fibers crossing crack surfaces, diminishing crack-bridging effects. Consequently, despite the increased fiber content, the fracture performance exhibits certain degradation.

On the other hand, when the PVA fiber content remains constant, *f*_R,1_ also changes with the increase in *a*_0_/*D*. Specifically, when the initial crack height increases from 2 cm to 5 cm, the maximum increase rate of *f*_R,1_ reaches 52.2%, and the average increase rate is 36.8%. This phenomenon can be attributed to the fact that an increase in *a*_0_/*D* makes it more likely for the fibers to cross the crack propagation process, forming an effective bridging effect, thus dissipating more energy and slowing down the crack propagation speed. However, when the initial crack-height ratio further increases (exceeding 0.5), due to size limitations, it is difficult for the fibers to connect the cracks again, and *f*_R,1_ may decrease accordingly.

#### 3.3.3. Discussion of Concrete Scratch and Softening Relationship

**Figure 12:**
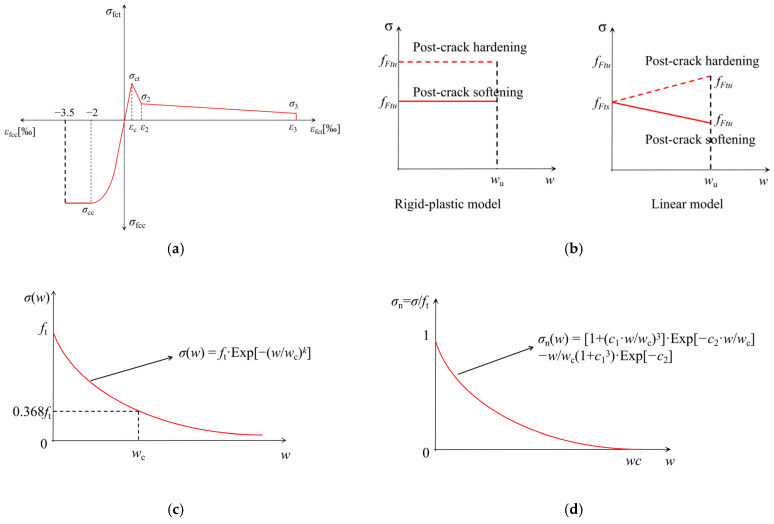
The scratch and softening models within the context of fiber reinforcement concrete (FRC). (**a**) The stress–strain distribution of FRC bending cross-section [36]. (**b**) Simplified post-cracking constitutive laws: stress-crack opening [23]. (**c**) Distributed crack model [44]. (**d**) Hordijk stress–crack width theoretical model [45].

The fracture behavior of concrete fundamentally depends on the mechanical characteristics of its fracture zone [41,42], where the tensile softening model (*σ*-*w* relationship) serves as an effective analytical tool for characterizing fracture-induced softening phenomena [29,43]. The scratch test methodology, employing displacement-controlled protocols, enables precise quantification of surface damage progression in fiber-reinforced concrete systems while simultaneously determining critical interfacial parameters (bond strength *τ* and crack width *w*_c_), thereby facilitating reliable *σ*-*w* relationship derivation for macroscopic fracture prediction. Figure 12 presents several typical concrete scratch test models and softening models. RILEM TC 162-TDF [36] proposed the scratch test model based on residual strength; Model Code 2010 [23] proposed the rigid-plastic linear stress–crack width softening model for the tensile zone; FIB Bulletin 103 [44] referenced the distributed cracking model for softening relationships; Hordijk’s research [45] studied nonlinear softening relationships in fiber-reinforced concrete, and Mi et al. [46] developed an inverse analysis algorithm that considers local response effects on *σ*-*w* curves while balancing model accuracy, computational cost, and generalizability. Therefore, establishing scratch models based on scratch tests and developing softening models through three-point bending tests remain key innovative directions for future research.

## 4. SEM Microstructural Analysis

**Figure 13:**
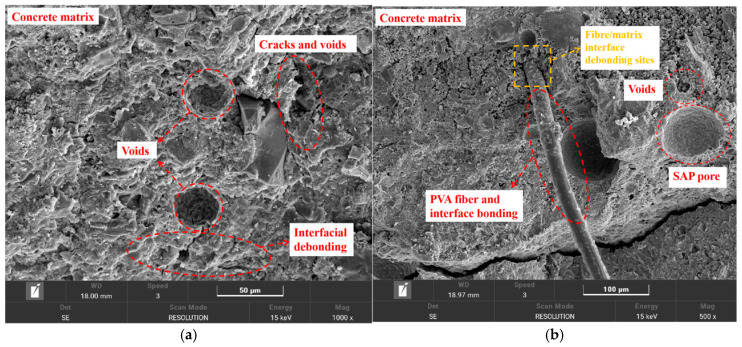

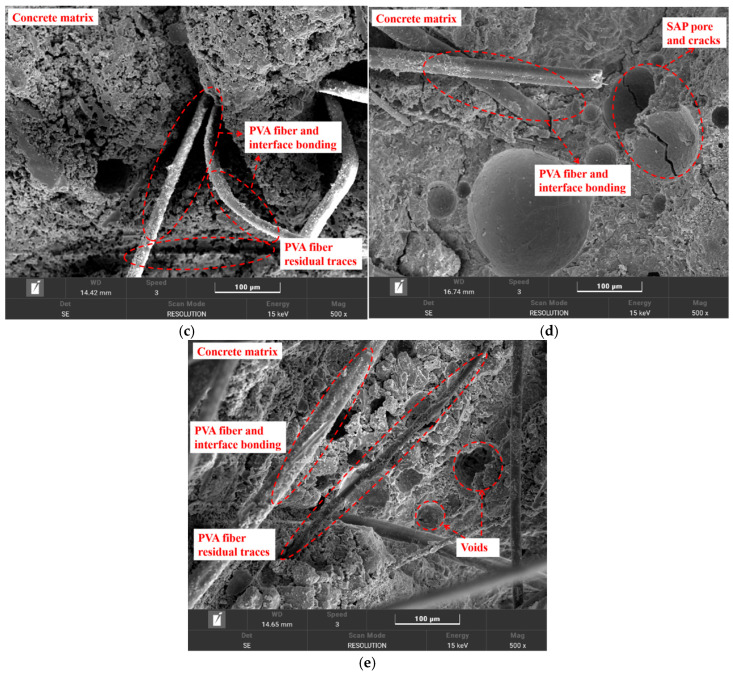
SEM micrographs of SAP-PVAC at different PVA volume content. (**a**) C. (**b**) SP-0.05%. (**c**) SP-0.10%. (**d**) SP-0.15%. (**e**) SP-0.20%.

Figure 12 shows the SEM morphology of SAP-PVAC specimens. It can be seen from the figure that the incorporation of SAP and PVA fibers objectively leads to an increase in the number of matrix pores, promoting the expansion of original micro-cracks and other defects inside the concrete. From Figure 13a–e, as the amount of SAP incorporated and the PVA fiber content increase, the bonding part between the fiber and the interface becomes more obvious. At this time, based on the mechanical and fracture test results of SAP-PVAC specimens (Figure 8, Figure 9 and Figure 11), the synergistic bridging effect of SAP and PVA fibers effectively suppressed interface debonding and separation, thus delaying the brittle fracture behavior typical of conventional concrete.

From the SEM pictures in Figure 13a, the traces of interface separation between the aggregate and the matrix under external load can be clearly observed, which intuitively shows the internal situation of interface fracture. In Figure 13b–e, it can be observed that the PVA fibers are closely wrapped with the concrete matrix, and the hydration products adhere to the surface of the PVA fibers, which significantly enhances the bonding force between the fibers and the matrix. However, at the microscopic level, the slip–debonding phenomenon between the root of the PVA fiber and the concrete matrix is also observed, as shown in Figure 13b. This demonstrates that PVA fibers can synergistically interact with SAP during concrete hydration to form chemical bonding products (such as hydrogen bonds, surface hydrates on PVA fibers, and other chemically bonded compounds). These interfacial bonding enhancements effectively strengthen the cohesion among fibers, matrix, and aggregates, thereby suppressing the propagation of micro-cracks and ultimately increasing the concrete’s fracture energy (as shown in Figure 9b).

Therefore, from a microscopic perspective, the synergistic effect of SAP and PVA fibers exhibits a positive promoting effect on the toughening and crack resistance of SAP-PVAC specimens.

## 5. Conclusions

This study carried out three-point bending tests on SAP-PVA fiber-reinforced concrete notched beams with different PVA fiber contents and initial crack-height ratios (*a*_0_/*D*) and analyzed the mechanical and fracture properties. Based on the macroscopic fracture phenomenon and the microscopic SAP-PVA fiber coupling mechanism, the toughening and crack-resistance effects of the SAP-PVA fiber synergy on concrete were comprehensively discussed, and the following conclusions could be safely drawn:

(1) The failure mode of SAP-PVAC specimens was significantly influenced by PVA fibers. At a higher fiber content, the open cracks of the specimens under load are smaller and more compact, and the crack propagation angle is also larger. The initial crack depth has no effect on the crack width at the time of specimen fracture, while the crack propagation angle of specimens with a larger *a*_0_/*D* is relatively smaller.
(2) The *P*-*CMOD* curves exhibited substantial variations in shape. Specimens with higher PVA fiber content demonstrated a maximum 14.3% increase in load-bearing capacity, accompanied by gentler post-peak softening behavior due to fiber reinforcement. Larger *a*_0_/*D* significantly reduced the specimens’ load-bearing capacity and failure deformation by an average of 22%, while simultaneously increasing the crack mouth opening displacement (*CMOD*) at critical states.
(3) The PVA fiber content exhibited dominant control in the range of 0.10–0.15%, significantly enhancing the fracture energy, fracture toughness, and residual tensile strength of concrete. In contrast, the *a*_0_/*D* showed negligible effects on fracture energy and toughness. However, analytical results revealed that variations in the *a*_0_/*D* could remarkably increase the residual flexural tensile strength (maximum enhancement of 33.0%), suggesting the probable existence of a threshold value for the *a*_0_/*D*.
(4) SEM-based microstructural analysis revealed that while SAP and PVA fibers objectively increased the incidence of initial defects in the concrete matrix, their synergistic coupling bridging effect enhanced interfacial bonding properties, demonstrating a positive role in toughening and crack resistance for SAP-PVAC specimens. However, this study has limitations regarding the characterization of microscopic fracture mechanisms in these specimens. Further research is required to quantify the micro-interfacial properties of SAP-PVAC composites, such as through nanoindentation or spectroscopic imaging techniques.

## Figures and Tables

**Table 1:** Parameters of super-absorbent polymer (SAP) particles [22].

Diameter (μm)	Density (kg/m^3^)	Water Absorption Rate (Pure Water)/%	Water Absorption Rate (9% NaCl)/%	Water Absorption Rate in 1 min (g/g)	pH	Water Content (%)
60~100	700	350	45	230	6~7	≤7

**Table 2:** Parameters of polyvinyl alcohol (PVA) Fibers [22].

Diameter (μm)	Length (mm)	Density (g/cm^3^)	Tensile Strength (MPa)	Initial Elastic Modulus (GPa)	Breaking Elongation (%)
15.09	12	1.29	1830	40	6.9

**Table 3:** Mix proportions of concrete specimens [22].

Specimen Item	Water (kg/m^3^)	Cement (kg/m^3^)	Sand (kg/m^3^)	Coarse Aggregate (kg/m^3^)	Water Reducer (kg/m^3^)	SAP (kg/m^3^)	PVA (kg/m^3^)	Water-Cement Ratio (kg/m^3^)	Internal Conservation Water Quality (kg/m^3^)
C	155	480	640	1160	1.2	0.000	0.000	0.323	0.0
SP-0.05%	155	480	640	1160	1.2	1.116	0.645	0.323	27.9
SP-0.10%	155	480	640	1160	1.2	1.116	1.290	0.323	27.9
SP-0.15%	155	480	640	1160	1.2	1.116	1.935	0.323	27.9
SP-0.20%	155	480	640	1160	1.2	1.116	2.580	0.323	27.9

Notes: C represents regular concrete (control group), SP-*p*%-*a*_0_/*D* represents internally SAP cured PVA fiber-reinforced concrete, *p*% denotes PVA fiber content.

**Table 4:** *P*_ini_ and *CMOD*_ini_, *P*_max_ and *CMOD*_c_, *a*_c_, and *E* of SAP-PVAC.

Specimen Item	*CMOD*_ini_ (mm) (SD)	*P*_ini_ (kN) (SD)	*CMOD*_c_ (mm) (SD)	*P*_max_ (kN) (SD)	*E* (GPa) (SD)	*f*_t_ (MPa) (SD)	*a*_c_ (mm) (SD)
C-0.133	0.007 (0.001)	10.01 (1.362)	0.029 (0.006)	17.11 (1.052)	32.06 (2.994)	3.545 (0.207)	42.81 (3.261)
C-0.200	0.011 (0.002)	8.78 (1.081)	0.038 (0.006)	15.34 (0.830)	34.01 (2.978)	3.550 (0.192)	48.73 (2.347)
C-0.333	0.013 (0.002)	6.44 (1.002)	0.055 (0.001)	10.99 (0.495)	36.46 (0.140)	3.663 (0.165)	71.44 (3.985)
SP-0.05%-0.133	0.008 (0.001)	9.92 (1.051)	0.029 (0.003)	16.95 (0.514)	29.41 (1.702)	3.343 (0.101)	33.08 (3.278)
SP-0.05%-0.200	0.011 (0.001)	8.79 (1.517)	0.044 (0.001)	14.66 (1.383)	26.83 (2.401)	3.394 (0.145)	49.33 (3.623)
SP-0.05%-0.333	0.012 (0.003)	4.88 (0.438)	0.054 (0.001)	10.94 (0.903)	29.94 (1.306)	3.378 (0.301)	69.36 (1.539)
SP-0.1%-0.133	0.008 (0.002)	10.14 (1.296)	0.028 (0.002)	19.55 (1.029)	29.64 (3.108)	3.622 (0.203)	38.62 (3.531)
SP-0.1%-0.200	0.011 (0.002)	9.45 (1.371)	0.044 (0.005)	15.60 (1.000)	33.50 (2.055)	3.575 (0.200)	56.93 (2.571)
SP-0.1%-0.333	0.030 (0.004)	6.94 (0.616)	0.069 (0.003)	11.24 (0.797)	31.09 (2.691)	3.465 (0.266)	74.32 (3.832)
SP-0.15%-0.133	0.010 (0.001)	11.68 (1.379)	0.036 (0.005)	18.83 (0.861)	26.82 (1.319)	3.576 (0.170)	37.74 (3.344)
SP-0.15%-0.200	0.012 (0.001)	9.34 (1.297)	0.043 (0.003)	16.51 (1.335)	26.66 (2.650)	3.486 (0.297)	47.82 (2.231)
SP-0.15%-0.333	0.019 (0.005)	6.82 (0.672)	0.058 (0.007)	11.52 (0.570)	28.48 (2.048)	3.638 (0.190)	67.66 (4.729)
SP-0.2%-0.133	0.008 (0.001)	10.94 (0.505)	0.033 (0.007)	18.37 (0.394)	33.10 (2.474)	3.623 (0.078)	41.07 (1.707)
SP-0.2%-0.200	0.010 (0.000)	8.68 (0.534)	0.038 (0.004)	15.24 (0.705)	31.11 (0.698)	3.527 (0.224)	48.35 (2.360)
SP-0.2%-0.333	0.014 (0.002)	6.14 (0.672)	0.053 (0.008)	10.91 (0.503)	36.06 (2.884)	3.637 (0.168)	71.57 (3.796)

Notes: C represents regular concrete (control group), and SP-*p*%-*a*_0_/*D* represents internally SAP cured PVA fiber-reinforced concrete, where *p*% denotes PVA fiber content, and *a*_0_/*D* denotes initial crack-height ratio. SD is the standard deviation.

**Table 5:** The fracture toughness, fracture energy, and residual tensile strength of SAP-PVAC specimens.

Specimen Item	$K_{IC}^{ini}$ (MPa·m^1/2^) (SD)	Standard Deviation (*a*_0_/*D*)	$K_{IC}^{un}$ (MPa·m^1/2^) (SD)	Standard Deviation (*a*_0_/*D*)	*G*_F_ (N/mm) (SD)	Standard Deviation (*a*_0_/*D*)	*f*_R,1_ (MPa)
C-0.133	0.580 (0.073)	0.033	1.490 (0.089)	0.058	0.241 (0.010)	0.021	0.635 (0.014)
C-0.200	0.531 (0.076)		1.391 (0.081)		0.271 (0.017)		0.737 (0.002)
C-0.333	0.518 (0.064)		1.494 (0.113)		0.230 (0.020)		0.828 (0.079)
SP-0.05%-0.133	0.540 (0.056)	0.061	1.185 (0.024)	0.104	0.237 (0.014)	0.025	0.686 (0.078)
SP-0.05%-0.200	0.586 (0.078)		1.363 (0.063)		0.210 (0.026)		0.750 (0.232)
SP-0.05%-0.333	0.466 (0.048)		1.367 (0.138)		0.188 (0.018)		0.862 (0.159)
SP-0.1%-0.133	0.594 (0.070)	0.032	1.503 (0.132)	0.100	0.264 (0.017)	0.009	0.826 (0.131)
SP-0.1%-0.200	0.629 (0.069)		1.703 (0.094)		0.274 (0.014)		0.983 (0.035)
SP-0.1%-0.333	0.657 (0.057)		1.614 (0.188)		0.281 (0.018)		1.258 (0.161)
SP-0.15%-0.133	0.634 (0.074)	0.012	1.373 (0.044)	0.037	0.266 (0.017)	0.011	0.912 (0.079)
SP-0.15%-0.200	0.622 (0.072)		1.360 (0.121)		0.278 (0.014)		0.988 (0.133)
SP-0.15%-0.333	0.646 (0.062)		1.430 (0.061)		0.287 (0.018)		1.222 (0.094)
SP-0.2%-0.133	0.595 (0.027)	0.008	1.479 (0.115)	0.078	0.272 (0.015)	0.010	0.809 (0.095)
SP-0.2%-0.200	0.579 (0.051)		1.390 (0.051)		0.253 (0.006)		0.843 (0.039)
SP-0.2%-0.333	0.583 (0.062)		1.545 (0.062)		0.267 (0.008)		1.120 (0.107)

Notes: C represents regular concrete (control group), and SP-*p*%-*a*_0_/*D* is internally SAP-cured PVA fiber-reinforced concrete, *p*% denotes PVA fiber content, and *a*_0_/*D* denotes initial crack-height ratio. SD is the standard deviation.

## Data Availability

The original contributions presented in this study are included in the article. Further inquiries can be directed to the corresponding author.
